# Supplementary figures and images for: Comprehensive catalog of dendritically localized mRNA isoforms from sub-cellular sequencing of single mouse neurons
Source: BMC Biol. 2019 Jan 24;17:5. doi: 10.1186/s12915-019-0630-z (PMC6344992; doi:10.1186/s12915-019-0630-z)

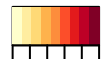

0 1  
Fraction  
dendritic

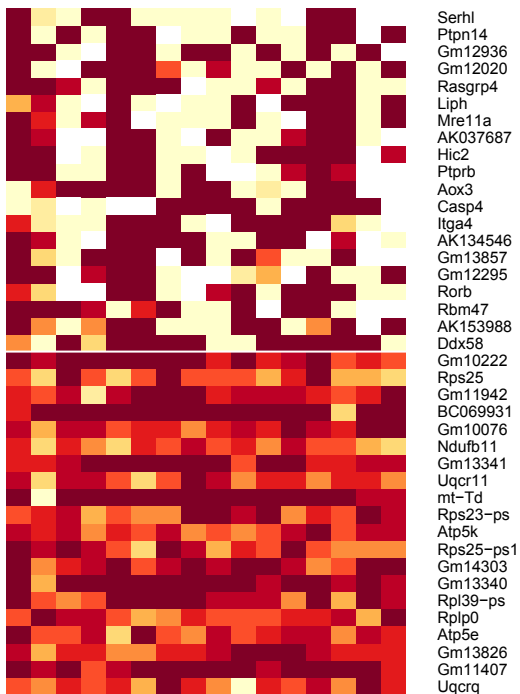

Cells

Supplement: Supplementary file 2 — Heatmap of subsampled variability of localization, related to Fig. 2d. The low variability genes were subsampled to 10 reads, the high variability genes are displayed as their original values. (PDF 222 kb) [file 12915_2019_630_MOESM2_ESM.pdf]
