# Supplementary material for: Comprehensive catalog of dendritically localized mRNA isoforms from sub-cellular sequencing of single mouse neurons
Source: BMC Biol. 2019 Jan 24;17:5. doi: 10.1186/s12915-019-0630-z (PMC6344992; doi:10.1186/s12915-019-0630-z)
Supplement: Supplementary file 3 — Re-analysis of differential localization of 3′UTR isoforms using 250 nt merge distance. (PDF 78 kb) [file 12915_2019_630_MOESM3_ESM.pdf]

Additional File 3: Re-analysis of differential localization of 3'UTR isoforms using 250nt merge distance.

To assess the impact of merging 3’UTRs within 500nt of each other, we repeated our isoform analysis using a smaller merge distance/quantification window of 250nt. This resulted in 3,574 multi-UTR genes being analyzed after filtering. 319 genes were found to have significant differential localization, with 216 overlapping with the 500nt window set (listed below). The characteristics of this set were almost identical to the 500nt set: dendrites preferred the distal isoform in 66.8% of cases, 62.4% of the genes had a more variable distal fraction in the soma, and the dendrite-preferred isoforms were significantly longer on average than the soma-preferred isoforms.

Overlapping genes between 250nt and 500nt window sets:

2410004B18Rik, 6430548M08Rik, A530058N18Rik, A830018L16Rik, AC149090.1, Aak1, Abhd16a, Abi1, Acly, Acss2, Actg1, Ahcyl2, Amdhd2, Amfr, Amph, Ank2, Ankfy1, Anp32e, Ap2a2, Ap3b2, Apbb2, Arfgap1, Arid1a, Arid2, Arl1, Atp5a1, Atp5f1, Atp5g1, Atp5h, Atp6v1b2, Atxn7l3b, BC005537, Bcl11a, Bdh1, Bex1, Bloc1s1, Bsg, Btf3, Calm3, Camk2b, Cbx5, Ccdc47, Ccl27a, Ccndbp1, Cd99l2, Cdc123, Cdc42, Cetn2, Chmp3, Cnot6l, Commd7, Copg1, Cops6, Csnk1d, Csnk2b, Cxxc4, Dhdds, Dhx30, Dos, Drg1, Dync1i2, Dynll2, E2f6, Ehmt2, Eif2ak1, Emc4, Emc7, Esf1, Evi5l, Fam171a1, Fam229b, Farsa, Fbxo31, Fbxo44, Fgd4, Flot2, Fscn1, Glud1, Gm14204, Gm15459, Gnai1, Gnb1, H2afy, Hdac5, Hnrnpu, Hsd17b12, Hsp90aa1, Hspa8, Ift57, Inpp4a, Itpa, Jtb, Kalrn, Kcnq2, Kpna1, Lamtor2, Ldha, Lsm3, Lysmd4, Maged2, Map1lc3b, Map2, Map2k4, Mapk8ip2, Megf11, Mettl2, Mfap3l, Minos1, Mkln1,

Mllt11, Mlx, Mpc1, Mrpl10, Mrpl52, Mrps23, Mrps35, Mtch1, Nav2, Ncam1, Ndrg4, Ndufa10, Ndufa9, Nsg2, Nudt21, Nudt3, Nxf1, Ociad1, Ociad2, Ogdh, Olfm1, Oxct1, Paf1, Paip2, Pank1, Papolg, Pccb, Pcgf5, Pcmt1, Pcmtd1, Pcna, Pdrg1, Peg3, Pgk1, Pigk, Pja2, Plcb1, Pmpcb, Polr2m, Ppdpf, Ppid, Ppm1h, Ppp3cb, Prpf38b, Psma6, Psmb2, Psmc4, Ptprd, Purg, Rab11fip2, Rab4b, Rac1, Ranbp1, Rbms3, Rpl15, Rpl31, Rpl5, Rtfdc1, Rufy3, Sap30l, Schip1, Scoc, Sdha, Sec14l1, Sec24a, Selk, Sept11, Sept2, Shisa5, Skp1a, Slc25a11, Slc25a3, Slc25a51, Slc25a5, Slc4a3, Slmo1, Snap91, Snrpb, Snx27, Spag9, Srp72, Srrm1, Stk39, Suclg1, Syt11, Taf11, Tbc1d14, Tbcel, Tfg, Tmem126a, Tmem59, Tpm3, Tsnax, Ube2e3, Ube2i, Ube2j2, Ubfd1, Uck2, Unc5c, Vapb, Vma21, Vps45, Wasf3, Wsb2, Yif1b, Znrf1, mt-Rnr2
