# Supplementary material for: Comprehensive catalog of dendritically localized mRNA isoforms from sub-cellular sequencing of single mouse neurons
Source: BMC Biol. 2019 Jan 24;17:5. doi: 10.1186/s12915-019-0630-z (PMC6344992; doi:10.1186/s12915-019-0630-z)
Supplement: Supplementary file 4 — Expanded structure analysis of potential locally translated proteins. Table S1. Predicted transmembrane structures. Table S2. Predicted RNA-binding structures. Table S3. Predicted structures commonly found in synaptic proteins. (PDF 189 kb) [file 12915_2019_630_MOESM4_ESM.pdf]

Additional file 4: Expanded structure analysis of potential locally translated proteins.

Membrane-bound

There were 274 proteins in our local proteome set with at least one high-confidence TM domain prediction (Table S1), including well known gated ion channel fold members such as Gria1/2, Grin1/2b, Kcnh7, and Scn2a1. The most common co-occurring folds with transmembrane folds included immunoglobulin-like beta-sandwiches (40 occurrences), which encompasses many cell adhesion structures such as cadherin; SH3-like barrels (29 occurrences), which includes many protein-protein interaction structures; and protein kinase-like structures (11 occurrences). Overall, these results support the idea that there are numerous locally-translated membrane proteins, which are likely translated on-demand during L-LTP to help stabilize the growing synapse, anchor intracellular scaffolds, and increase signal transduction through the synapse.

RNA binding

RBPs are known to play crucial roles in localizing RNAs to the dendrites and in regulating their translation, but there is little known about whether RBPs are locally translated themselves. There were 138 proteins in our local proteome set with a high confidence match to an RNA-binding fold (Table S2) and 77 with a medium confidence match, demonstrating that a wide variety of RBPs may indeed be produced by local translation. Among these were important neuronal RBPs such as Atxn2, Stau1/2, Elavl2/3, Mbnl2, and Cpeb2. In addition, several of the predicted RBPs either were not previously known to be RBPs, or were known to bind RNA but did not yet have an annotated RBD. Two examples of the latter category were Dync1h1

(Cytoplasmic dynein 1 heavy chain 1), for which we predicted a Poly(A) binding protein (PABP) domain-like structure between residues 2,042 and 2,174; and Trub2 (Probable tRNA pseudouridine synthase 2), which we predicted to have a OB-nucleotide binding domain between residues 40 and 86, adjacent to the known catalytic domain. Looking into the medium-confidence predictions, we also found completely novel RBP predictions such as Mga (MAX gene-associated protein), a transcription factor that we predicted to have a dsRBD-like fold (residues 563-862) downstream of the DNA-binding domain; and Akap11 (A-kinase anchor protein 11), a kinase-regulating protein that we predict to have a type I KH-domain fold at the C-terminal (residues 1,501-1,894).

Synaptic functions

The PDZ fold is one of the most well-characterized protein structures involved in the synapse because of the crucial role it plays in protein-protein interactions between the intracellular scaffolding of the spine and membrane-bound receptors as well as cell adhesion molecules [14]. There were 21 proteins in the local proteome set that contained at least one PDZ fold, with many containing more than one (Table S3). All 21 of these proteins were previously annotated as containing a PDZ domain by Gene3D, indicating that this fold has already been well characterized across proteins. Similarly, all eight of the predicted guanylate kinase (GK) domains and all 32 of the predicted SH3 domains—both of which frequently co-occur with PDZ domains at the synapse [15]—were previously annotated (Table S3). These results highlight the potential role of local translation as a source for these important scaffolding proteins.

Many other synapse-related folds had a mixture of both known and novel predictions. We predicted two new domains with a Pleckstrin homology (PH) domain, Nischarin (Nisch) and

Sphingosin kinase 2 (Sphk2). Although both of these proteins are known to be phosphatidylinositol-binding, the location of the PH domain was not yet annotated. Another novel prediction was made for Capicua (Cic), a transcriptional repressor that interacts with Ataxin-1 and plays a role in central nervous system development. We predicted this protein to have a previously-unannotated Tudor domain near its N-terminal. Tudor domains may play a role in stress granule formation through binding of methylated RGG motifs [16] and more generally are found in RNPs. This suggests potential new roles for Capicua beyond its known transcription-related functions. We highlight additional known and novel predictions for membrane-bending Bin-Amphiphysin-Rvs (BAR) domains and actin-binding Calponin homology (CH) domains in Table S3.

Table S1. Local proteome: predicted transmembrane structures.

SCOP	Structure name	Predicted proteins
f.1	Toxins' membrane translocation domains	Bcl2l2, Wdfy3*
f.3	Light-harvesting complex subunits	Bnip3l*, Ntrk3*
f.13	Class A G protein-coupled receptor (GPCR)-like	Atp6v0a1*, Gabbr1*, Gpr162, Lgr5, Oprd1, Svop
f.14	Gated ion channels	D3Bwg0562e, Gabrb3, Gria1, Gria2, Grin1, Grin2b, Hcn1, Kcnh7, Kcnq5, Ndfip1*, Scn2a1, Scn8a
f.17	Transmembrane helix hairpin	Acsl6*, Ankfy1*, Atp5g1, Atp5g2, Atp5g3, Atp6v0e2*, Atp9a*, Cadm1*, Canx*, Cd84*, Chrdl1*, Emc4*, Epha6*, Ern1*, Gbp7*, Gm15487, Higd1a*, Higd2a*, Kcna1, Kcna2, Kcng3, Kcnq5, Krtcap2*, Lman2*, Lpgat1*, Mdga2*, Ppp2r5b*, Ptprd*, Rnf5*, Romo1*, Sec62*, Slc3a2*, Slitrk5*, Tmem14c*, Tmem167*, Tmem256*, Tmem258*, Ube2j2*, Ugt1a6a*, Vma21*, Vps35*
f.19	Aquaporin-like	Aqp4, Palm
f.21	Heme-binding four-helical bundle	Agtrap*, Kcnq2, Sdhc, Sdhd, Slc22a15, Slc4a3*, Tmem170b*, Tmem50b*
f.23	Single transmembrane helix	AI413582*, AY036118*, Abhd6*, Acsl4*, Ahcyl1, Anapc5*, Aplp2*, Arel1*, Armcx1*, Armcx2*, Atp1a3*, Atp1b1, Atp2a2*, Atp5j2*, B3gat1*, B3gat2*, Bcl2l2*, Bdnf*, Bsg*, Caly*, Ccpg1*, Cd84*, Cd99l2*, Cdadc1*, Cdh13*, Celf2*, Celf4*, Cend1*, Chd3*, Chd4*, Chp1, Chst2*, Clec2l*, Clip3*, Cnot6l*, Cntn1*, Comt*, Coro1c*, Cox4i1, Cox6a1, Cox6a2, Cox6c, Cox7a2, Cox7a2l, Cox7b, Cox7c, Cox8a, Crlf2*, Crtac1*, Csf2ra*, Cyb5*, Cyb5b*, Dlc1*, Dner*, Egf*, Elavl2*, Elmo1*, Enpp5*, Epha5*, Epha6*, Erbb4, Exo1*, Fam115a, Fam155a*, Fam174a*, Flrt2*, Foxp2*, Gabrb2*, Gabrg2*, Gdap1*, Gli3*, Gltpd2*, Gria1*, Gria2*, Grin3a*, Herc1*, Herc2*, Hsd17b12*, Hspa5*, Huwe1*, Ids*, Ier3ip1*, Itga1, Itga4*, Kcna1, Kcna2, Kcng3, Kcnq2*, Kcnq5, Klf9*, Lman2*, Lrrc4b*, Lrrc4c*, Lsamp*, Lypd1*, Macf1*, Mavs*, Mdga2*, Megf11, Mfap3l*, Mia3*, Mkrn1*, Mpc1*, Mpc2*, Mrpl9*, Myo5a, Ndufa1*, Ndufa4*, Ndufa9*, Ndufb2*, Ndufb3*, Ndufb8*, Ndufc1*, Ndufc2*, Nenf*, Nlgn1*, Nlgn2*, Nrxn1*, Nrxn2*, Nrxn3*, Ntrk2*, Ntrk3*, Opcml*, Pam*, Pcmtd1*, Pdgfrl*, Pigk*, Pitpnm1*, Plin3*, Pnkd*, Ppm1h*, Ppp2r5b*, Psd*, Ptprb*, Ptprs*, Pum2*, Pvrl3*, Rbm47, Rhot1*, Rnf130*, Robo2*, Rps2*, Rtn2*, Scn2a1*, Sec11c*, Sel1l*, Selt*, Serp2*, Serpina3k*, Sez6l2*, Slc22a15*, Slc25a12, Slc25a23*, Slc30a9*, Slc4a3, Slco1a1*, Slitrk5*, Smdt1*, Smim13*, Sparc*, Sparcl1*, Spock2*, Srl*, Synj2bp*, Syt15*, Tef*, Tmx4*, Tnrc6a*, Tomm20*, Tomm6*, Tor4a*, Tsc22d2*, Tusc3*, Txndc15*, Ubqln2*, Ugt1a6a*, Ulk2*, Unc5c*, Uqcr10, Uqcr11, Uqcrfs1, Uqcrq, Usmg5*, Usp34*, Wdfy3*, Xpo7*, Zeb2*
f.27	14 kDa protein of cytochrome bc1 complex (Ubiquinol-cytochrome c reductase)	Uqcrb

f.28	Non-heme 11 kDa protein of cytochrome bc1 complex (Ubiquinol-cytochrome c reductase)	Uqcrh
f.32	a domain/subunit of cytochrome bc1 complex (Ubiquinol-cytochrome c reductase)	Grin3a*
f.35	Multidrug efflux transporter AcrB transmembrane domain	Disp2, Ptchd4
f.42	Mitochondrial carrier	Gda, Slc25a11, Slc25a12, Slc25a22, Slc25a23, Slc25a3, Slc25a4, Slc25a5, Slc25a51
f.45	Mitochondrial ATP synthase coupling factor 6	Atp5j*
f.49	Proton glutamate symport protein	Slc1a1, Slc1a2
f.51	Rhomboid-like	Slc17a9, Slc22a15, Slc22a17, Svop
f.53	ATP synthase D chain-like	Atp5h*, Gm10250*, Sptbn2
f.56	MAPEG domain-like	Abca5*, Cnih2*, Kcng3, Mgst3, Rabac1*, Sc4mol*, Timm17a*, Timm17b*
f.57	MgtE membrane domain-like	Disp2, Slc28a3*
f.58	MetI-like	Abca5*, Atp9a*, Mboat7*, Mmd*, Slc17a7, Slc23a1*, Slc28a3*, Slc2a13, Slc7a11*, Sv2a, Tlcd1*
f.59	Cation efflux protein transmembrane domain-like	Slc30a9

* new annotation (compared to Gene3D)
All predictions shown are high confidence (nearest neighbor distance ≤ 17.5)

Table S2. Local proteome: predicted RNA-binding structures.

Fold	Desc	Predicted proteins
a.144	PABP domain-like	Dync1h1*, Pabpc1
a.217	Surp module (SWAP domain)	Zc3h7b*
b.38	Sm-like fold	Atxn2, Lsm3, Lsmd1, Snrpb, Snrpn
b.40.4	OB-fold; Nucleic acid binding	Ccdc141, Cmip, Csdc2, Csde1, Dlst, Dnaaf2*, Eif5a, Gm10263, Pdgfrl, Polr2g, Polr3h, Rapgef4, Rpl6, Rps11, Rps23, Rps28, Trub2*, Ttc14, Ybx1, Zcchc17
d.265	Pseudouridine synthase	Rpusd4, Trub2
d.41	alpha/beta-Hammerhead	Aox3, Mocs2, Rpl10
d.50	dsRBD-like	Adarb1, Dhx9, Rps2, Stau1, Stau2
d.51	Eukaryotic type KH-domain (KH-domain type I)	Fubp1, Hnrnpk, Pcbp2
d.58.7	Canonical RNA binding domain (RBD) [RRM]	Alyref, Celf2, Celf4, Cnot4, Cpeb2, Cpsf6, Eif4h, Elavl2, Elavl3, Ewsr1, Fus, G3bp2, Hnrnpa1, Hnrnpa2b1, Hnrnpa3, Hnrnpab, Hnrnpm, Msi2, Ncbp2, Ncl, Nxf1, Pabpc1, Pabpn1, Ppargc1a, Ppargc1b, Rbfox1, Rbfox2, Rbm14, Rbm17, Rbm25, Rbm47, Rbms3, Slirp, Syncrip, Tnrc6a, Uhmk1, Zcrb1
g.66	CCCH zinc finger	Mbnl2, Mkrn1, Rc3h1, Rc3h2, Zc3h15, Zc3h7b

* new annotation (compared to Gene3D)
All predictions shown are high confidence (nearest neighbor distance ≤ 17.5)

Table S3. Local proteome: predicted structures commonly found in synaptic proteins.

SCOP	Structure name	Predicted proteins
b.36	PDZ domains	Apba1, Dlg2, Dlg4, Dvl1, Dvl3, Frmpd4, Gorasp2^, Grip1, Limk1, Lin7a, Magi1, Mast1, Mpp3, Ppp1r9b, Ptpn4, Rims1, Shank2, Shank3, Sipa1l1, Snx27, Synj2bp
c.37.1.1	Nucleotide and nucleoside kinases [includes GK]	Cacnb4, Cmpk1^, Dlg2, Dlg4, Hnrnpu^, Mpp3^, Ndufa10, Stxbp1^
b.34.2	SH3 domains	Abi1, Abi2, Amph, Arhgef4, Arhgef9, Bcar1, Cacnb4, Caskin1, Crk, Dlg2, Dlg4, Itsn1, Kalrn, Map3k10, Mapk8ip1, Mapk8ip2, Mcf2l, Mia3, Mpp3, Pacsin1, Rasa1, Rusc1, Sh3gl2, Sh3glb2, Shank2, Shank3, Sorbs2, Sptan1, Srgap3, Stam, Ubash3b, Vav3
b.55.1.1	PH domains	Abr, Adap2, Apbb1ip, Arap2, Arhgef4, Arhgef9, Cadps, Cdc42bpa^, Elmo1, Exoc8, Fgd4, Kalrn, Kif1a, Kif1b, Mcf2l, Nisch*^, Pdpk1, Psd, Rasa1, Sos2, Sphk2*^, Sptbn1, Sptbn2, Vav3
b.34.9.1	Tudor domains	A830010M20Rik*, Cic*, Slc25a12*, Trp53bp1
a.238	BAR domains	Amph, Appl1, Arfip2, Cog7*^, Dync1h1^, Exoc6b*^, Macf1*^, Mtss1l^, Pacsin1*^, Sh3gl2, Smarca2*^
a.40	CH domains	Camsap1, Ccdc88a*, Dmd, Macf1, Mapre1, Mapre2, Mical2, Nav2, Nav3, Parva, Parva^, Sptbn1, Sptbn2, Stxbp1^, Vav3

* new annotation (compared to Gene3D)
^ medium-confidence prediction (nearest neighbor distance ≤ 30); all others are high confidence (nearest neighbor distance ≤ 17.5)
