# Supplementary material for: Comprehensive catalog of dendritically localized mRNA isoforms from sub-cellular sequencing of single mouse neurons
Source: BMC Biol. 2019 Jan 24;17:5. doi: 10.1186/s12915-019-0630-z (PMC6344992; doi:10.1186/s12915-019-0630-z)
Supplement: Supplementary file 7 — Overview of 3′UTR definition, quantification, selection of top two isoforms, and calculation of distal fraction. (PDF 275 kb) [file 12915_2019_630_MOESM7_ESM.pdf]

1. Quantify 3'UTRs

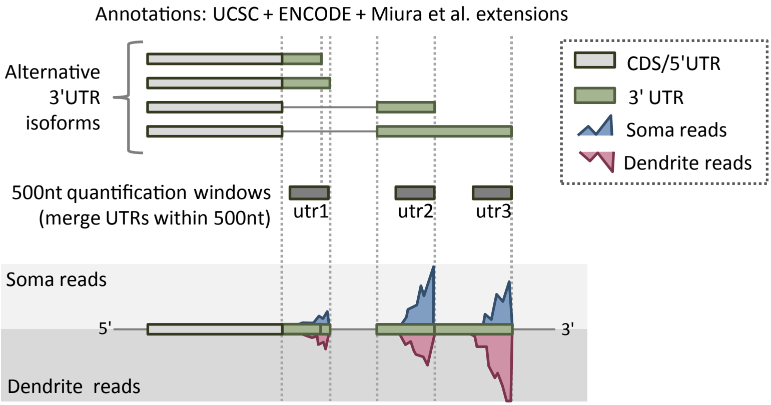

2. Identify top two isoforms

	utr1	utr2	utr3
Sample 1	0.02	0.32	0.66
Sample 2	0	0.29	0.71
...	...	...	...
Sample 32	0.04	0.25	0.71
Sum of fractions	0.5	10.3	21.2

Fraction of reads mapping to each 3'UTR window for each sample

Identify top two UTRs by summing fractions for each UTR over all samples with at least 10 reads

3. Calculate distal fraction (DF)

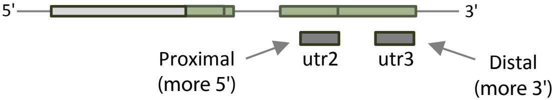

$$DF = \frac{\#\ Reads\ Distal}{\#\ Reads\ Distal + Proximal}$$
